# Optimizing pineapple production under waterlogged soil condition in low input management using adequate ridge tillage height and plant density

**DOI:** 10.3389/fpls.2025.1570261

**Published:** 2025-06-03

**Authors:** Georges Marius Etame Kossi, Honore Djonko Beyegue, Alexis Boukong, Rose Germaine Ossogo, Lionel Lidjo

**Affiliations:** ^1^ Department of Agriculture, Faculty of Agronomy and Agricultural Sciences, University of Dschang, Dschang, Cameroon; ^2^ Department of Soil Science, Faculty of Agronomy and Agricultural Sciences, University of Dschang, Dschang, Cameroon

**Keywords:** water management, soil tillage, sustainable intensification, *ananas comosus*, waterlogging

## Abstract

Pineapple production is greatly hindered by waterlogging. This condition reduces the profitability of pineapple producer in areas frequently under temporary or permanently waterlogging soil condition. This situation weakens pineapple production and accentuate poverty in rural small-scale pineapple family producers, imbalance diet of consumer and high failure risk for young entrepreneur engage in transformation and commercialization of pineapple fruits. This study investigates the potential of ridge height and planting density on the growth and yield of *Ananas comosus* in waterlogged soil conditions. A split-plot design was employed, featuring three levels of ridge height (15 cm, 30 cm, and 45 cm) and two planting densities (27,777 and 57,142 plants.ha^1^) with four replications. Data was collected on shoot and root at growth, yield formation and harvest stage. The results indicated that a ridge height of 30 cm significantly enhanced root development, leaf area, and fruit yield, achieving an increase of 149% in yield with crown compared to 15 cm ridges. A ridge height of 45 cm creates a capillary barrier approximately 15 to 20 cm below the top of the ridge, which alters water movement within the soil during both wet and dry periods. Additionally, increased planting density resulted in improved resource utilization without adversely affecting growth parameters. The highest fruit yield of 86.9 t.ha^-1^ was recorded at 30 cm ridge height with a density of 57,142 plants.ha^1^, demonstrating the potential of optimized agroecological practices in enhancing pineapple production in waterlogged conditions. Incorporating soil moisture sensors, as noted in recent studies, could optimize water management and prevent water stress, contributing to more stable yield outcomes. Appropriate ridge height and optimal plant density optimize resource use by pineapple plant in waterlogging soil condition.

## 1 Introduction

Pineapple (*Ananas comosus*) is an important fruit crop in the world, contributing to fight against hunger and imbalanced nutrition for over billions of people in world (Firatoiu et al., 2021). In the last ten years, the increasing production demonstrates the need for this crop in the human diet, with demand exceeding supply and the development of good market opportunities (Firatoiu et al., 2021). Therefore, in parts of the central region of Cameroon covered by forest, the increase in pineapple production has led to forest destruction due to the expansion of cropping areas (Etame Kossi et al., 2023b). Whereas forest-savannah transition area is suitable for pineapple without reduction of forest surface, which preserves against adverse effects such as climate change, loss of biodiversity, and soil erosion (Etame Kossi et al., 2023a).

Pineapple was first introduced and cultivated in the forest-savannah transition zone of the Centre Region in Cameroon. From there, its cultivation gradually expanded into the forest zones of both the Centre and Littoral Regions. Pineapples grown in the forest-savannah transition zone are particularly favored by consumers due to their taste quality. This zone covers nearly 50% of the Centre Region, according to the Ministry of Territorial Administration. Additionally, the area serves as a crossroads connecting several regions in Cameroon, which has facilitated the distribution of pineapples and other agricultural products, while also helping to reduce transportation costs.

In this area, pineapple producers face several challenges. The dominant gender in pineapple production are women, facing poor financial conditions, weak labor capacity, limited knowledge of good cultivation practices, especially in terms of plant density, and environmental constraints such as soil waterlogging (Etame Kossi et al., 2023a, 2023b). All these challenges lead these resilient women to abandon pineapple cultivation. The abandonment of pineapple cultivation in this area increases pineapple prices in metropolitan areas, heightens the risk of failure for many young entrepreneurs involved in fruit transformation and commercialization, and exacerbates dietary deficiencies for the least privileged segments of the population.

Waterlogging is a condition in which soil becomes saturated with water, although there is no water visible on the soil surface. When water does appear on the soil surface, this condition is referred to as flooding (Tian et al., 2021). Waterlogging limits pineapple growth and development, increases the length of the growing cycle, and reduces fruit weight and total yield (Cahyono et al., 2018; Tian et al., 2021). In extremely adverse conditions, no marketable fruit is harvested. Waterlogging reduces oxygen availability to plant roots, limiting their capacity to absorb water and nutrients and increase plant susceptibility to soil born pathogens (Tyagi et al., 2024). This restriction decreases photosynthetic activity, resulting in lower production and translocation of essential compounds to various plant organs, ultimately stunting plant growth and development (Min and Bartholomew, 2002; Beegum et al., 2023). Additionally, suboptimal pineapple plant density leads to lower yields and extensively use of land resources (Valleser, 2018; Djido et al., 2021; Neri et al., 2021). Although, the planting density in forest areas typically ranges from 30,000 to 60,000 plants.ha^-1^. However, the actual planting density utilized in forest-savannah transition zone is considerably lower, with sole cropping systems using fewer than 25,000 plants.ha^-1^ and intercropping systems using fewer than 10,000 plants.ha^-1^ (Etame Kossi et al., 2023b). This planting density is substantially lower than the recommended range of 55,000 to 75,000 plant.ha^-1^ (Djido et al., 2021; Neri et al., 2021). Taking into consideration all the challenges faced by small pineapple producers in the forest-savannah transition areas of the Centre region of Cameroon, how can effective agroecological management alleviate waterlogging and improve pineapple production?

The work of Manik et al. (2019) highlights ridging as an effective agroecological management practice for draining soil under waterlogged conditions without requiring significant financial resources. Ridge formation is more than just elevating soil to a specific height; as ridge height increases, compaction tends to intensify, creating a capillary barrier that restricts capillary rise and limits water infiltration within the ridge profile. This leads to two main impacts: in the rainy season, high moisture at the ridge top may limit plant growth, while in the dry season, capillary rise is restricted to 25 cm, reducing water availability, particularly for crops with shallow root systems (Ma et al., 2024). To optimize ridge height, it is essential to consider both the depth of the water table during the growing period and the crop’s sensitivity to waterlogged conditions (Tay, 1974; Ma et al., 2024). Nevertheless, there is no recommendation regarding the optimal height of ridges needed to ensure the success of this practice and optimize pineapple production under waterlogged conditions. Also, to intensified pineapple production, it is essential to adopt an appropriate plant density. Proper plant spacing can increase yield by approximately 40–71% without compromising fruit weight (Djido et al., 2021; Neri et al., 2021). However, no specific planting density is recommended for pineapple producers across the agroecological zones of Cameroon.

This study aims to define the optimal ridge height for effective waterlogging management in pineapple production and, secondly, to analyze the contribution of increased pineapple planting density to yield. There exists at least one ridge height and planting density that improve resources uses and increase pineapple yield in waterlogged soil condition.

## 2 Materials and method

### 2.1 Location of study area

**Figure 1:**
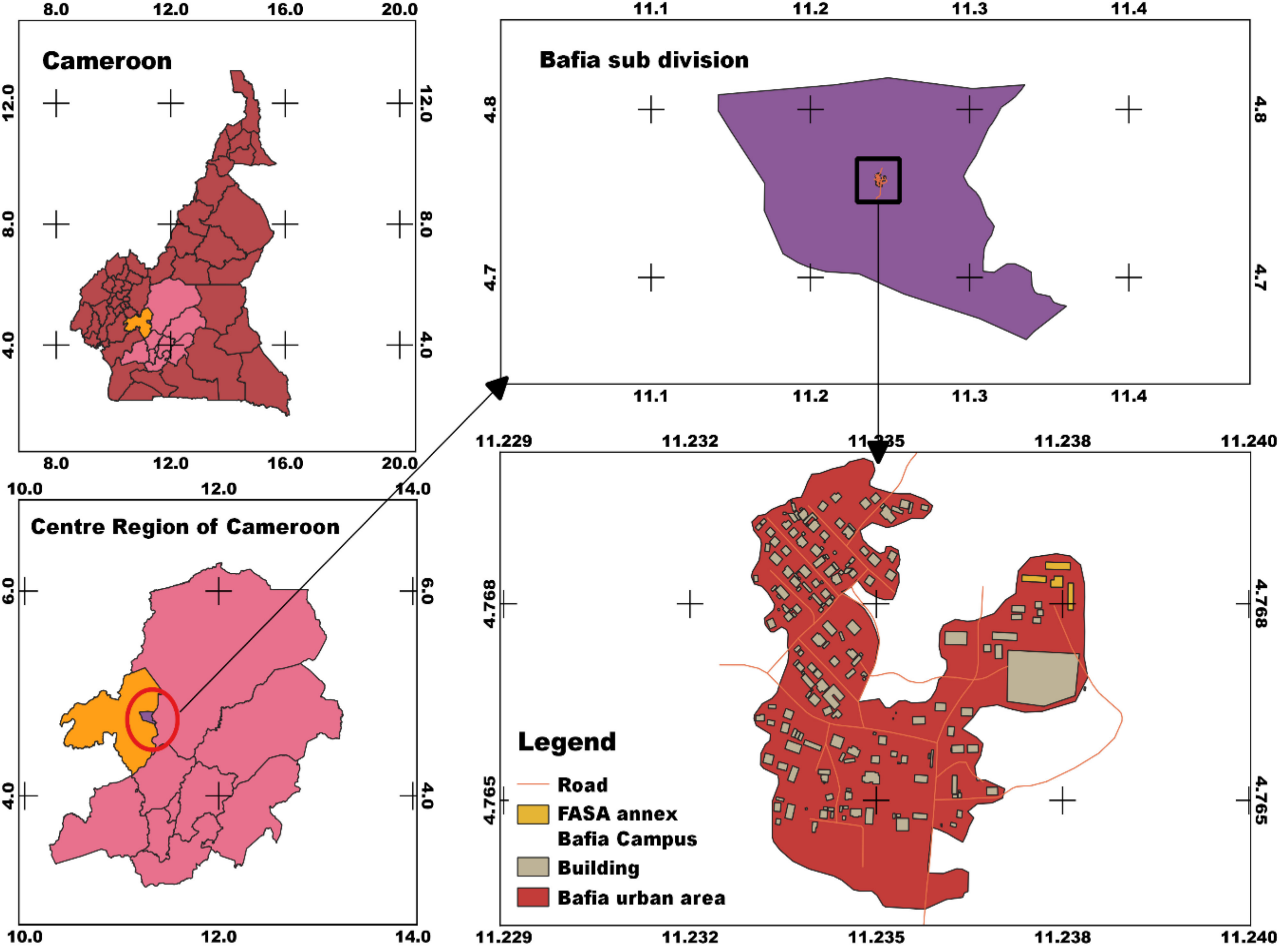
Location of experimental site.

The commune of Bafia is located in the Centre region of Cameroon (**Figure 1**), within a forested agroecological zone characterized by bimodal rainfall patterns. Bafia is situated specifically at coordinates 4.60° N latitude and 11.240° E longitude, with an elevation ranging from 500 to 700 meters above sea level. This area experiences an annual average rainfall of 1,300 to 1,400 mm and an average annual temperature of 25°C (Jagoret et al., 2012). The main soil types encountered are Acrisols, making up nearly 80% of the soils in the Mbam and Inoubou department. These Acrisols exhibit Dystric characteristics, and sometimes Gleyic. The remaining 20% consists of Dystric Nitisols. These soils are low in major nutrients such as nitrogen, phosphorus, and/or potassium, are slightly to moderately acidic, and are dominated by kaolinite (Vallerie, 1973; Jones et al., 2015).

### 2.2 Experimental design

A split-plot design was conducted at the Research and Application Farm of FASA, Annex of Bafia, to examine the effects of ridge height and planting density on pineapple growth. This factorial experiment included ridge height as the primary factor, with three levels (15, 30, and 45 cm), and planting density as the secondary factor, with two levels 27,777 (80×45 cm) and 57,142 plants.ha^1^ (80×40×35 cm). The Smooth Cayenne pineapple variety served as the test crop sowed at 05 June 2023. Experimental units of 6 m² were set up in quadruplicate, with 50 cm spacing within each block and 1 m spacing between blocks, covering a total area of 423.5 m². Surrounding the main trial, two rows were planted with a spacing of 80 × 45 cm, equivalent to a planting density of 27,777 plants.ha^1^, under a zero-tillage method to serve as an observation plot.

### 2.3 Management practice

Pineapple was planted on June 7 using suckers weighing between 450 and 500 g at 10 cm depth. The planting material was immersed in a fungicide and insecticide mixture for 15 minutes, then allowed to dry in ambient air for one day before planting. Weeding was carried out three times: the first manually with a hoe two months after planting, and the second and third using a chemical herbicide (diuron 800 g/kg) applied at a rate of 200 mL per 15-liter sprayer at 5 and 8 months after planting. Fertilization was performed once, using a 14-24–14 formulation at a rate of 7 g per plant, when symptoms of phosphorus deficiency appeared five months after planting. Floral induction was carried out using 200 ml of ethephon per 15-liter sprayer, to which 75 g of urea was added to increase the efficacy of floral induction. 15 ml of this solution was applied to the plant rosette when the plants had between 41 and 50 active leaves, as recommended by Barker et al. (2018).

### 2.4 Data collection

Data were collected on five plants randomly selected within each experimental unit and the observation plots surrounding the experimental units. Data collection started 60 days after planting (DAP) and ended 240 DAP, done each 30 days interval, which represents 15 days before floral induction. Variables collected included plant height, the number of active leaves, and the length and width of the D-leaf. Based on these variables, leaf area and plant vigor index were estimated using Equations 1, 2, as given by Dos Santos et al. (2018) and Padonou et al. (2019), respectively.


(1) LAID=−214,12+(2,938×LDL)+(7,329×WDL)

where LAID is the leaf area of the D-leaf in cm², LDL is the length of the D-leaf in cm, and WDL is the width of the D-leaf in cm.


(2) PVI=LDL×NAL

where PVI is the plant vigor index, LDL is the length of the D-leaf, and NAL is the number of active leaves.

**Figure 2:**
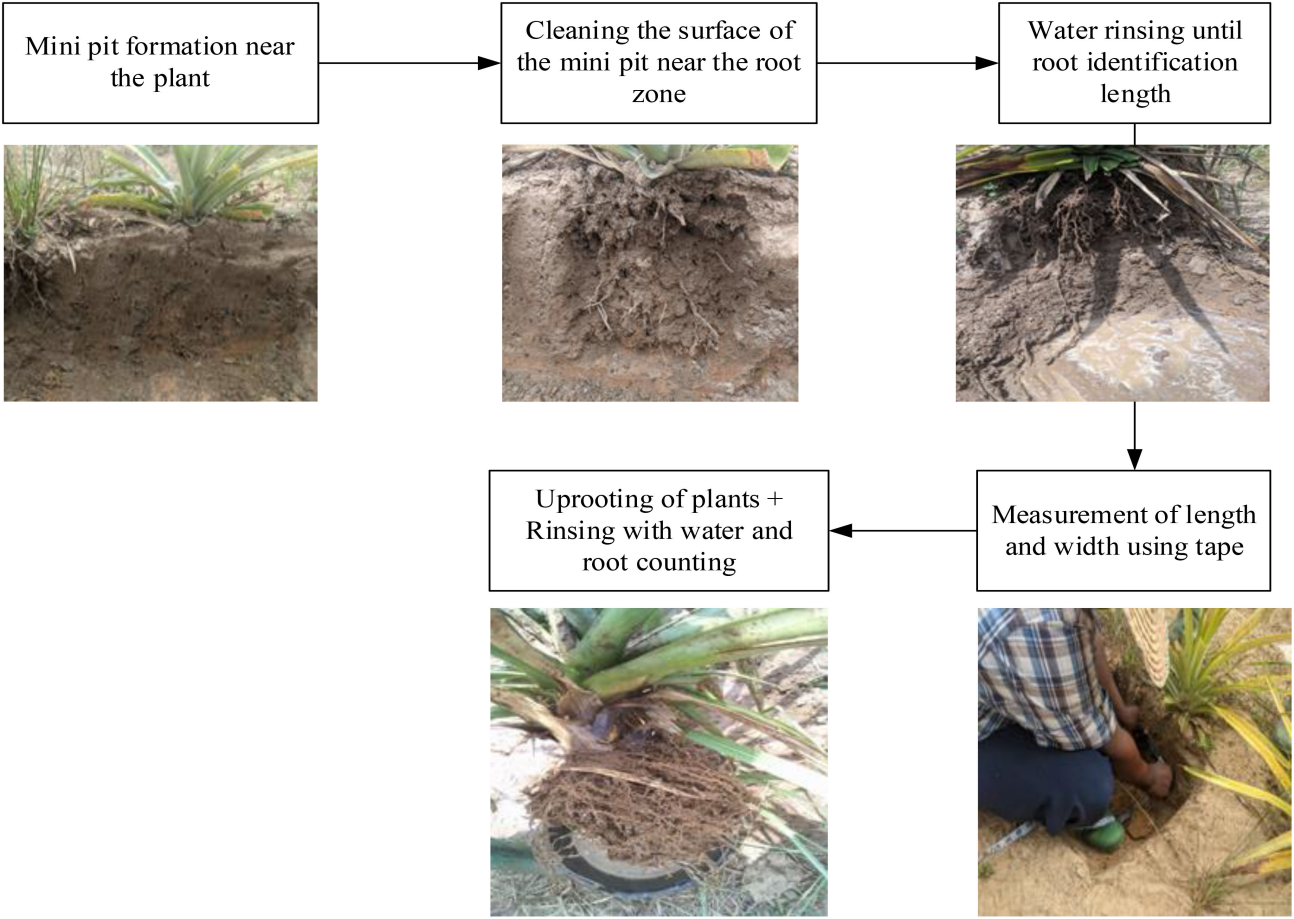
Root data collection step.

Fifteen days after floral induction, root development was observed *in situ* on three plants within each unit and observation plot. Observed variables included root length (RoLe) and width (RoWi), soil volume explored by roots (SoVo), root count (RoNo), average root diameter (RoDi). The three plants were then uprooted, roots washed, and counted (**Figure 2**). This was performed five days after floral induction.

### 2.5 Harvesting and yield estimation

Fruits were harvested 141 days after floral induction, 410 DAP (days after planting) from the five sampled plants used for growth data collection. Each fruit was cut using a knife and weighed with a scale. Collected variables included the fruit weight with and without the crown. Yield (Ye) was estimated using Equation 3 below:


(3) Ye=AFW×PD

where Ye is the yield in kg/ha, AFW is the average fruit weight in g (with or without the crown), and PD is the planting density (plants/ha).

*Data processing and statistical analysis*

The collected data were processed using Microsoft Excel 2019 and analyzed with R software version 4.3.2. Parametric test conditions, specifically normality (Shapiro test) and homogeneity of variances (Levene’s test), were verified using the RcmdrMisc package (Fox et al., 2023), before performing ANOVA (Analysis of variance) follow by Tukey test for separation of means with ExpDes package (Ferreira et al., 2014).

### 2.6 Evaluation of ridge height and planting density profitability

The profitability analysis of the tested technologies was conducted using the Benefit-cost ratio (BCR) and profitability (PR). BCR measures the profitability of adopting a new technology minus the costs associated with its implementation. The calculation of VCR followed the steps below:

Determining Additional Soil Work Cost (ASWC) (Equation 4):


(4) ASWC=HLC×TR×NR

where ASWC is the additional soil work cost; TR is the time needed to make a ridge on 100 m² (6.5 hours for 15 cm high, 12.5 hours for 30 cm high, and 19.5 hours for 45 cm high ridges); NR is the number of ridges per hectare (67 ridges of 100 m length, 1 m width, spaced 0.5 m apart); HLC is the hourly labor cost, set at 256 CFA (based on an 8-hour workday for 22 days a month with a 45,000 FCFA monthly wage, the minimum wage in the agricultural sector in Cameroon**).**

Determining Additional Plant Density Cost (APDC) (Equation 5):


(5) APDC=NAP×CP

where APDC is the additional plant density cost, NAP is the number of additional plants, and CP is the cost per plant, including transport and planting (set at 25 FCFA).

Determining Additional Harvesting Cost (AHC) (Equation 6):


(6) AHC=NAF×UFHP

where AHC is the additional harvesting cost, NAF is the number of additional fruits, and UFHP is the unit fruit harvesting price (2 CFA).

Calculating Total Investment Cost (TIC) (Equation 7):


(7) TIC=ASWC+APDC+AHC

Determining Additional Harvest Revenue (AHR) (Equation 8):


(8) $$AHR=\left(\frac{YeRnDn-YeA}{AFWRnDn}\right)\times FPRnDn$$

where AHR is the additional harvest revenue, YeRnDn is the fruit yield at ridge height n and density n; YeA is the zero-tillage fruit yield; AFWRnDn is the average fruit weight at ridge height n and density n; and FPRnDn is the fruit price based on ridge height n and planting density n, with H1 = 15 cm, H2 = 30 cm, and H3 = 45 cm for ridge heights and D1 = 27,777 plants.ha^1^ and D2 = 57,142 plants.ha^1^. The retained selling price was 125 FCFA for grade G (fruit weight between 1400–1599 g) and 75 FCFA for grade H (fruit weight between 800–999 g).

Calculating Investment Return Cost (IRC) (Equation 9):


(9) IRC=TIC+(TIC×a)

where IRC is the investment return cost, TIC is the total investment cost, and a is the investment interest rate set at 4.25% per annum.

Calculating Benefit-Cost Ratio (BCR) (Equation 10):


(10) BCR=AHR/IRC

Profitability (PR) (Equation 11):


(11) PR=BCR−1

where PR represents profitability.

### 2.7 Soil characteristics of the study site

The soil at the experimental site is deep and neutral, with a pH of 7.1. It is rich in calcium and magnesium but extremely deficient in potassium, phosphorus, and total nitrogen. The soil shows slight compaction, with a bulk density of 1.3 g/cm³, and is low in organic matter, evidenced by a favorable decomposition rate, represented by a C/N ratio of 11.1. Additionally, it has an acceptable base saturation level. To support optimal growth and yield of pineapple, supplementation of nitrogen, phosphorus, and potassium (N, P, K) nutrients is recommended (**Table 1**).

**Table 1:** Soil characteristics of experimental site.

Cl	Si	Sa	Nt	OC	OM	Da	EC	pHw	pHkcl	ΔpH	C/N	CEC	Ca^++^	Mg^++^	K^+^	Na^+^	S	V	P
%						g/cm^3^	mmhos	1:2.5				meq/100g						%	ppm
13.5	21.0	65.5	0.1	0.9	1.5	1.3	0.0	7.1	6.1	-1.0	11.1	23.4	7.2	3.5	0.0	0.6	11.4	48.9	6.0

Sa, sand; Si, silt; Cl, clay; Nt, total nitrogen; OC, organic carbon; OM, organic matter; EC, electrical conductivity; pHw, water pH; C/N, carbon nitrogen ratio; CEC, cation exchange capacity at pH 7; Ca^++^, available calcium; Mg^++^, available magnesium; K^+^, available potassium; Na^+^, available sodium; S, exchangeable base; V, base saturation percentage; P, assimilable Bray II phosphorus.

## 3 Result

### 3.1 Correction of waterlogging and soil depth influencing the growth and development of pineapple

The formation of ridges significantly enhances pineapple growth from 150 days post-planting, with the optimal ridge height identified as 30 cm (**Table 2**). Plants cultivated on 30 cm ridges exhibited a larger D-leaf area, greater D-leaf width, and a higher leaf count. Conversely, although plants on 45 cm ridges displayed slightly greater height and D-leaf length than those on 30 cm ridges, these differences did not reach statistical significance (**Table 2**). Plants on 15 cm ridges demonstrated limited growth across all observed parameters (**Table 2**). Additionally, plants in zero-tillage plots surrounding the experimental area showed notably slow growth, with an average height of 35.2 cm, corresponding to growth reductions of 60.5%, 86.1%, and 90.9% when compared with plants on 15 cm, 30 cm, and 45 cm ridges, respectively. This trend was also evident for leaf count, D-leaf length, and width. Growth differentials between plants on 15 cm and 30 cm ridges were observed as 15.8%, 21.5%, 14.6%, and 23.9% increase for height, leaf count, D-leaf length, and D-leaf area, respectively, while differences between plants on 30 cm and 45 cm ridges were minimal, at 2.6%, -9.0%, 1.5%, and -3.8% for these same variables (**Table 2**).

**Table 2:** Effect of ridge height and plant density on growth and development of pineapple plant.

Source	60 DAP	90 DAP	120 DAP	150 DAP	180 DAP	210 DAP	240 DAP
Plant height							
Ridge height (cm)							
15	14.4 ± 1.4a	20.9 ± 2.1a	22.9 ± 2.2a	26.9 ± 4.7a	35.5 ± 5.8a	49,9 ± 5,0a	56,5 ± 4,9a
30	14.9 ± 1.0a	21.4 ± 2.3a	24 ± 2.2a	30.5 ± 4.7b	45.5 ± 7.3c	58,1 ± 3,7b	65,5 ± 5,0b
45	14.3 ± 2.0a	22.0 ± 2.1a	24.6 ± 2.3a	27.3 ± 5.0a	41.3 ± 6.9b	52,7 ± 8,3ab	67,2 ± 8,4b
p-values	0.45	0.62	0.41	0.01*	0.003**	0,01*	0,053
Plant density (plants.ha^-1^)							
27 777	14.1 ± 1.0a	20.7 ± 1.5a	23.1 ± 2.2a	26.8 ± 4.1a	39.7 ± 7.4	52,2 ± 6,8a	62,1 ± 7,9a
57 142	15 ± 1.9a	22.1 ± 2.5a	24.5 ± 2.3a	29.6 ± 5.6b	41.8 ± 8.2	54,9 ± 8,3a	64,2 ± 5,9a
p-value	0.14	0.12	0.058	0.07	0.052	0,19	0,063
H×D p-value	0.95	0.38	0.28	0.32	0.061	0,09	0,11
Active leaf number							
Ridge height (cm)							
15			22.1 ± 2.5a	28.8 ± 2.1a	38.6 ± 4.6a	34,2 ± 8,4a	41,7 ± 8,2a
30			24.41 ± 2.9a	31.8 ± 4.9a	40.9 ± 7.5a	41,6 ± 6,9b	50,7 ± 13,3a
45			22.4 ± 2.8a	28.3 ± 2.4a	35.7 ± 8.0a	36,6 ± 8,5ab	46,5 ± 9,1a
p-value			0.18	0.09	0.53	0.03*	0.14
Plant density(plants.ha^-1^)							
27 777			22.8 ± 2.4a	29.2 ± 3.3a	37.2 ± 8.2a	35,6 ± 8,0a	43,4 ± 8,4a
57 142 ^-1^			24.0 ± 3.7a	31.7 ± 4.7a	39.6 ± 5.6a	39,9 ± 8,1a	49,2 ± 12,7a
p-value			0.75	0.16	0.27	0.12	0.06
H×D p-values			0.79	0.51	0.18	0.36	0.07
Length of D leaf							
Ridge height (cm)							
15			40.5 ± 4.6a	46.3 ± 4.0a	48.7 ± 4.2a	49,5 ± 3,1a	57,2 ± 7,4a
30			44.6 ± 3.9a	45.2 ± 7.0a	56.7 ± 3.5a	57,6 ± 5,4b	65,6 ± 5,5b
45			45.5 ± 2.6a	49.3 ± 7.2a	55.3 ± 3.7a	59,1 ± 3,8b	66,6 ± 4,7b
p-values			0.13	0.24	0.06	0,02*	0,01*
Plant density (plants.ha^-1^)							
27 777			43.6 ± 4.3a	46.5 ± 6.3a	53 ± 4.7a	53,7 ± 5,4a	62,3 ± 8,3a
57 142			43.5 ± 4.4a	47.7 ± 6.7a	54.7 ± 5.0a	56,5 ± 6,5a	63,9 ± 6,4a
p-value			0.89	0.25	0.10	0.06	0.09
H×D p-value			0.28	0.52	0.38	0.09	0.06
D leaf width							
Ridge heigh (cm)							
15			3.6 ± 0.2a	3.6 ± 0.4a	4.8 ± 0.5a	4.8 ± 0.6a	5.3 ± 0.7a
30			4.0 ± 0.1b	4.3 ± 0.7b	5.6 ± 0.7a	5.7 ± 0.5b	6.4 ± 0.4a
45			4.0 ± 0.2b	4.1 ± 0.5ab	5.3 ± 0.4a	5.6 ± 0.3b	5.6 ± 0.2a
P-value			0.03*	0.03*	0.08	0.04*	0.06
Plant density (plants.ha^-1^)							
27 777			3.9 ± 0.3a	4.0 ± 0.6a	5.1 ± 0.4a	5.3 ± 0.6a	5.6 ± 0.6a
57 142			3.9 ± 0.3a	4.0 ± 0.6a	5.4 ± 0.8a	5.5 ± 0.6a	5.8 ± 0.6b
p-value			0.97	0.50	0.12	0.09	0.04*
H×D p-value			0.36	0.83	0.47	0.99	
Leaf area index of D leaf							
Ridge heigh (cm)							
15			177.0 ± 21.3a	189.4 ± 32.3a	287.9 ± 44.0a	293,7 ± 64,2a	344,7 ± 57,5a
30			217.3 ± 19.3b	234.0 ± 64.6a	369.5 ± 48.9b	376,5 ± 47,6ab	427,1 ± 43,2b
45			213.2 ± 23.3b	239.9 ± 54.6a	338.4 ± 29.0b	378,6 ± 29,2b	411,5 ± 23,7b
p-value			0.007**	0.09	0.03*	0.03*	0.02*
Plant density (plants.ha^-1^)							
27 777			202.6 ± 27.6a	217.3 ± 57.1a	318.0 ± 32.0a	333,9 ± 69,1a	384,9 ± 55,7a
57 142			202.5 ± 28.5a	225.0 ± 56.4a	350.0 ± 62.0a	361,4 ± 57,6a	404 ± 55,9b
p-value			0.98	0.29	0.07	0.07	0.008**
H×D p-value			0.51	0.74	0.49	0.98	0.64

* Significant at 5%; ** highly significant at 1%; DAP, days after planting; H×D: interaction between ridge height and planting density, a ± b: mean ± standard deviation.

**Figure 3:**
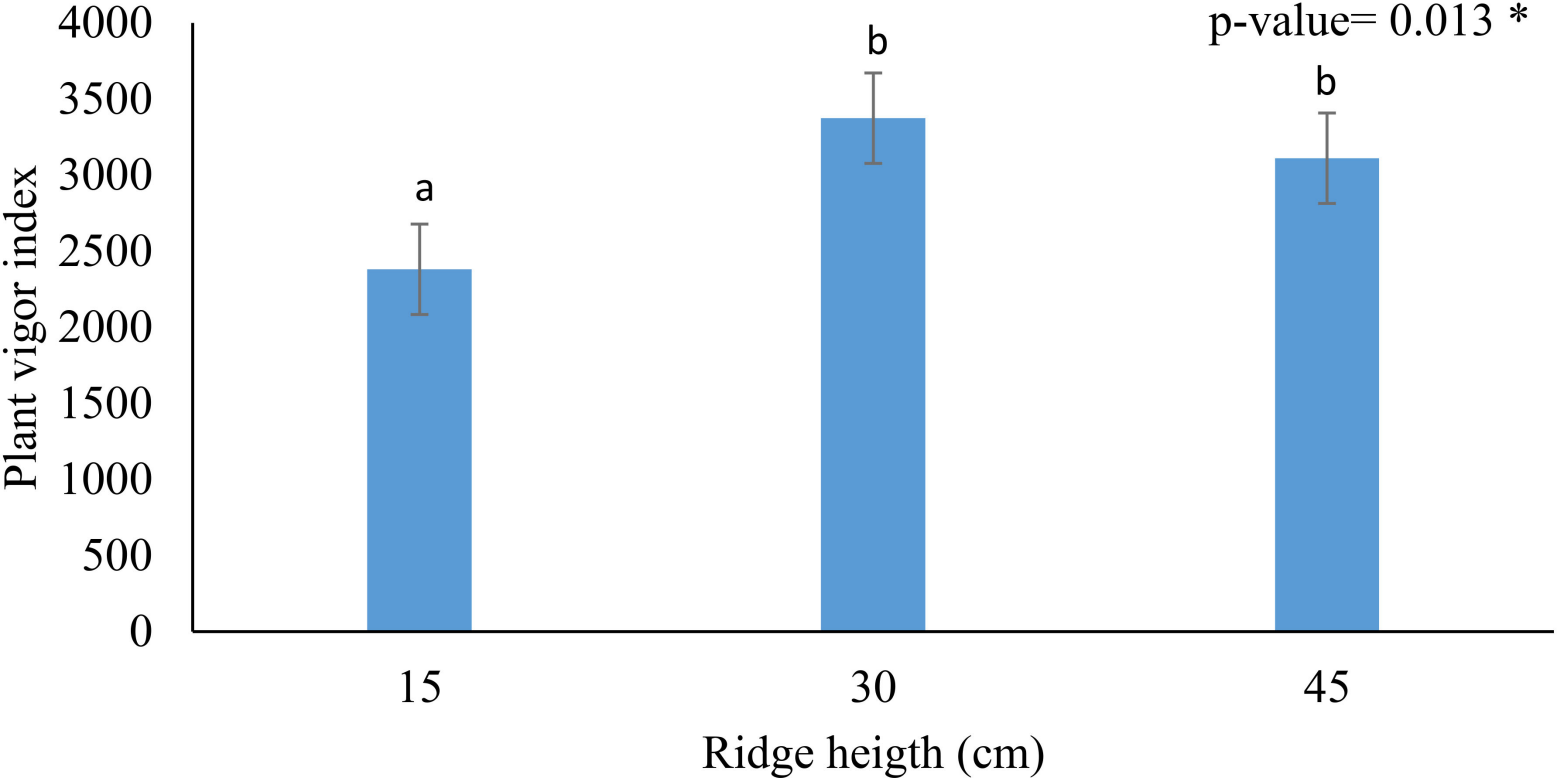
Effect of ridge height on plant vigor at floral induction. *: indicating significant difference. Same lowercase are not statistical different.

Given the time required to construct each ridge height 10, 20, and 30 minutes for 15, 30, and 45 cm ridges, respectively. There appears to be limited advantage to creating 45 cm ridges in the waterlogged soils of the Bafia-Bokito basin. The number of active leaves, a biological parameter influenced by genotype, varied significantly only at 210 DAP, but this parameter is slightly influenced by pedoclimatic factors and management type (**Table 2**). During floral induction, plant vigor peaked on 30 cm ridges, followed by 45 cm ridges, with vigor increase of 41.7% and 30.7%, respectively when comparing 15 cm ridges (**Figure 3**). At 240 DAP, plant height was highest on 45 cm ridges, though previous height values were comparable or lower than those of plants on 30 cm ridges. After prolonged drought, water retention also appeared higher in 45 cm ridges relative to 30 cm ridges; however, this trend may shift decreasing following floral induction and the return of seasonal rainfall, which typically introduces moist soil conditions.

**Figure 4:**
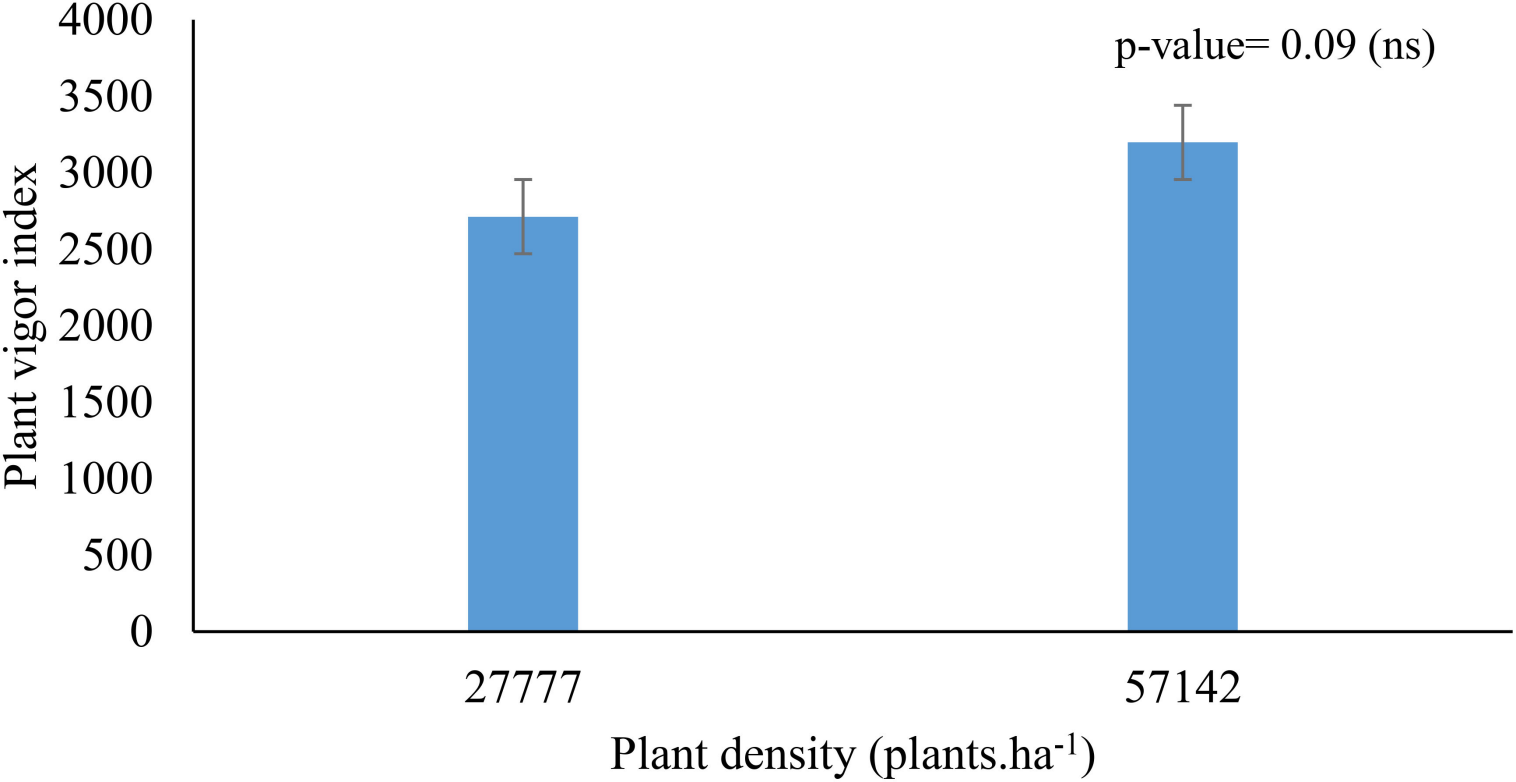
Effect of planting density on plant vigor.

Higher planting densities enhanced resource utilization by approximately 5% at 240 days post-planting, increasing D-leaf area by between 8.2% and 4.9% (**Table 2**). A vigor increase of 17.9% was observed between densities of 27,777 and 57,142 plants.ha^1^ (**Figure 4**).

### 3.2 Morphological pineapple root growth and development in waterlogged conditions is affected by ridge tillage

**Figure 5:**
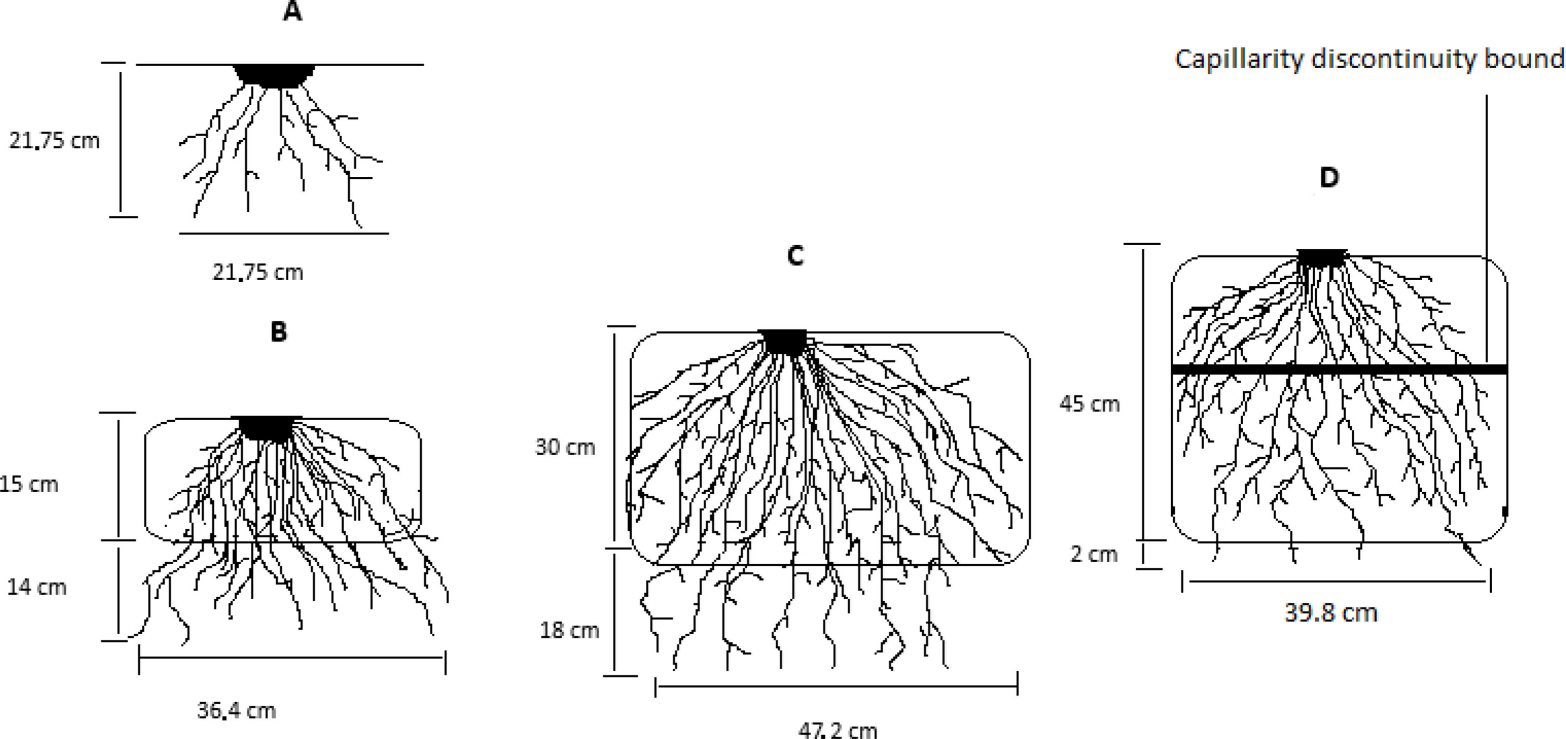
Effect of Ridge Heights on Root Development in Waterlogged Soil Conditions; **(A)** Root development under zero-tillage (direct-seeding control); **(B)** Root development on a 15 cm ridge; **(C)** Root development on a 30 cm ridge; **(D)** Root development on a 45 cm ridge.

Pineapple growth is impeded in conditions of waterlogging, exhibiting diminished root development with an average root length of 21.7 cm (**Figure 5**). Soil tillage has been demonstrated to enhance aeration and promote root development. Specifically, increasing ridge height has been observed to enhance root length, with a peak of 48.3 cm observed on 30 cm ridges, followed by 47.2 cm on 45 cm ridges, and 39.1 cm on 15 cm ridges. This

corresponds to increases of 122.5%, 117.5%, and 80%, respectively, relative to direct seeding. Additionally, soil ridging has been observed roots number increasing by 15.6%, 69.1%, and 35.2% on 15, 30, and 45 cm ridges height as compared to zero tillage, respectively. Nevertheless, ridge height does not exert a notable influence on root diameter (**Table 3**). The formation of ridges serves to mitigate the detrimental effects of waterlogging by establishing favorable conditions for aeration and moisture, thereby promoting the growth of pineapples. The optimal ridge height for pineapple cultivation in the soils of Mbam and Inoubou appears to be 30 cm, as this effectively enhances growth and development. The efficiency with which resources are utilized improves in tandem with increasing planting densities, as indicated by a 4.6% increase in root diameter.

**Table 3:** Effects of ridge height and planting density on root development.

Treatment	RoLe (cm)	RoWi (cm)	SoVol (dm^3^)	RoNo	RoDi (mm)
Ridge heigh (cm)					
15	39.1 ± 2.9a	36.4 ± 4.8a	54.9 ± 16.7a	64.4 ± 7.4a	1.13 ± 0.07a
30	48.3 ± 5.1b	42.7 ± 2.8b	86.7 ± 22.3b	80.3 ± 15.4b	1.12 ± 0.04a
45	47.2 ± 3.4b	39.8 ± 6.5ab	74.3 ± 24.3b	64.2 ± 7.4a	1.10 ± 0.05a
p-values	0.000**	0.03*	0.01*	0.02*	0.51
Plant density (plants.ha^-1^)					
27 777	44 ± 6.1a	40 ± 4.5a	72 ± 24.5a	67 ± 12.2a	1.09 ± 0.04a
57 142	46 ± 5.4a	39 ± 6.4a	72 ± 25.5a	73 ± 13.3a	1.14 ± 0.06b
p-values	0.54	0.76	0.95	0.31	0.01*
H×D p-values	0.36	0.45	0.08	0.87	0.46

*Significant at 5%** highly significant at 1%; DAP, days after planting; RoLe, root length; RoWi, root width; SoVo, soil volume explored by roots; RoNo, root count; RoDi, average root diameter; H×D, interaction between ridge height and planting density, a ± b, mean ± standard deviation.

### 3.3 Ridge tillage management practice optimize yield of pineapple under waterlogging condition

**Figure 6:**
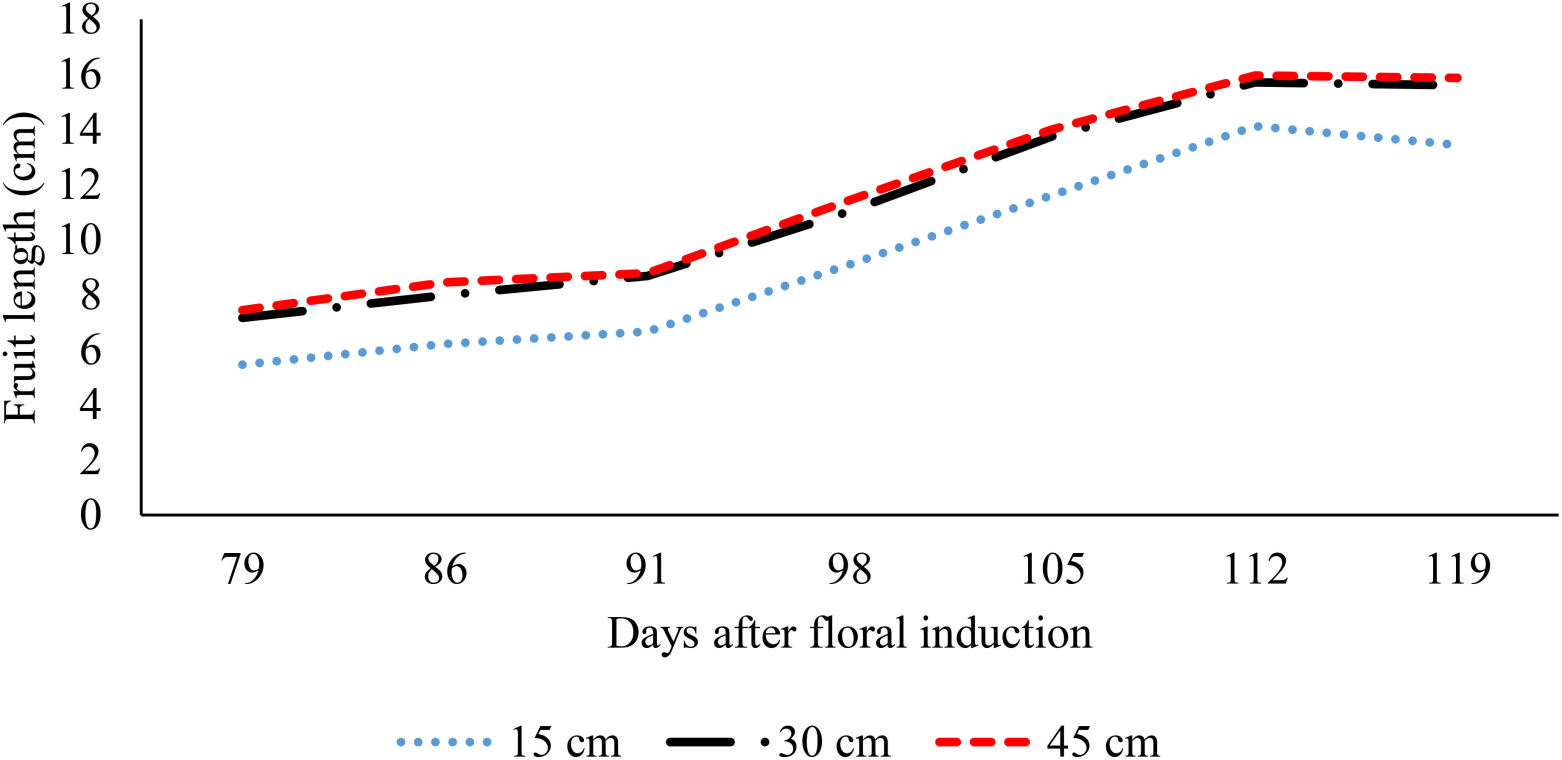
Effect of ridge height on fruit length.

**Figure 7:**
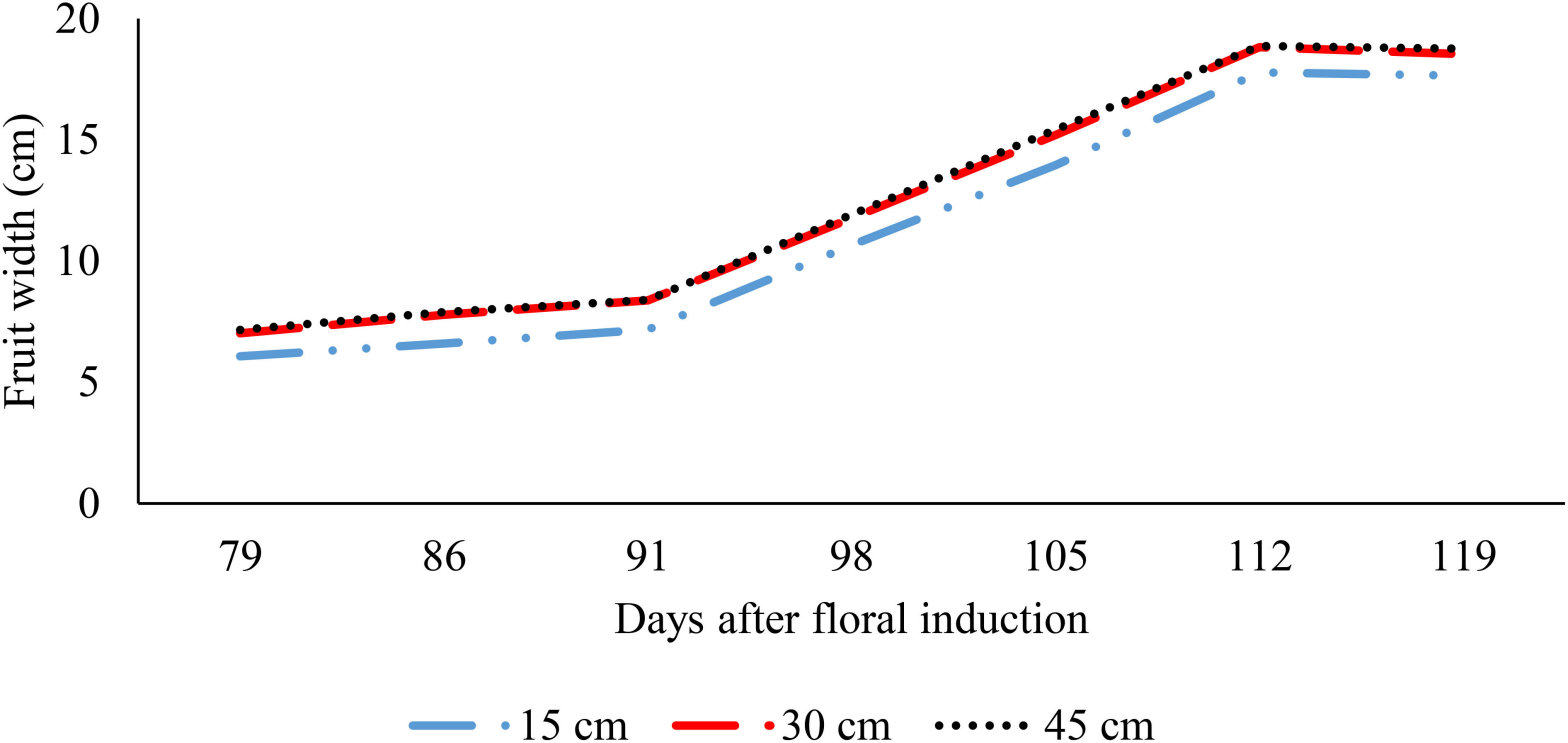
Effect of ridge height on fruit width.

**Figure 8:**
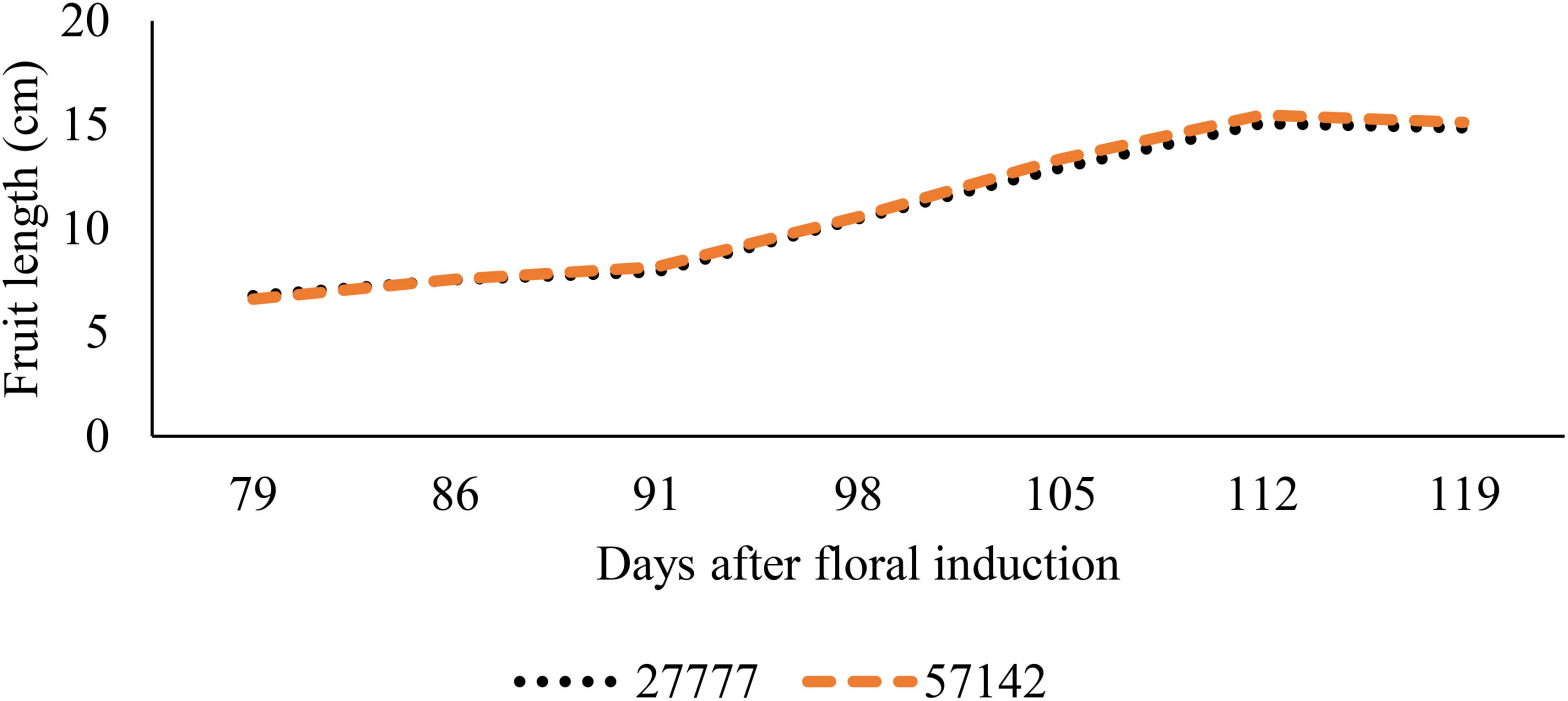
Effect of planting density on fruit length.

**Figure 9:**
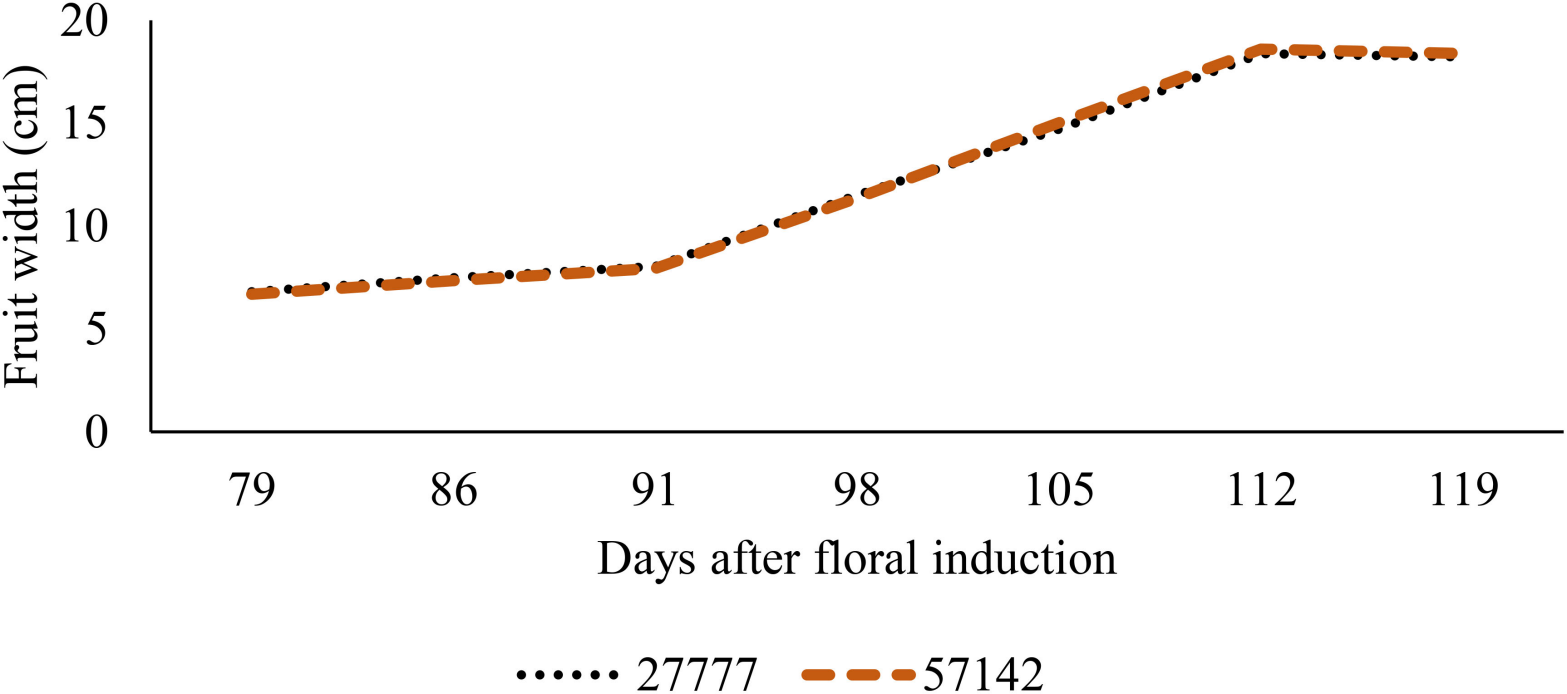
Effect of planting density on fruit width.

The process of ridge formation has also been demonstrated to promote fruit growth in situations where the soil is subjected to waterlogging (**Figures 6**, **7**). The fruit length increased by 28.9% and 33.5%, respectively, when grown on 30 cm and 45 cm ridges in comparison to 15 cm ridges; a further increase of 3.6% was observed between the 45 cm and 30 cm ridges (**Figure 6**). Similar trends were observed for fruit width, with increases of 16.1% and 17.9% for fruits grown on 30 cm and 45 cm ridges, respectively, relative to those on 15 cm ridges, and a 1.5% increase between 45 cm and 30 cm ridges (p< 0.05) (**Figure 7**). The impact of increased planting density on fruit parameters was inconclusive; mean fruit length and width remained consistent between densities of 27,777 and 57,142 plants.ha^-1^ (p > 0.05) (**Figures 8**, **9**).

The average fruit weight peaks at 1519 g per fruit on 45 cm ridges, with fruits on 30 cm ridges closely following at 1432 g. By contrast, fruits cultivated without ridging yield a significantly lower average weight of 527 g with crown and 270 g without. Cultivating pineapple on ridges of 30 to 45 cm in waterlogged soil conditions dramatically boosts fruit weight by 49% and 58%, respectively, compared to production on 15 cm ridges, and by a remarkable 171% and 188% relative to no-ridging planting. Even at 15 cm, ridges enhance fruit weight by 81%, adding 432 g compared to fruit weight on non-ridged soil. Ridging thus profoundly optimizes root aeration, water utilization, and nutrient availability under waterlogged conditions (**Table 4**). Moreover, increasing planting density from 27,777 to 57,142 plants per hectare elevates average fruit weight by 6%, ensuring that each pineapple plant utilizes water and nutrients to maximum efficiency. Fruits cultivated in waterlogged conditions without ridging fail to meet the quality standards of grades H to A as per Codex Standard 182-1993, rendering them non-viable for market. By contrast, increased ridge heights elevate fruit grade from H to E, enhancing market value from 75 to 125 FCFA per unit.

**Table 4:** Effect of ridge height and planting density on fruit weight with and without crowns.

Factors	Fruit mean weight in g	
	Without crown	With crown
Ridge height (cm)		
15	606 ± 42a	959 ± 50a
30	1047 ± 136b	1432 ± 125b
45	1136 ± 153b	1519 ± 117b
p-value	0,000***	0,000***
Plant density (plants.ha^-1^)		
27777	870 ± 234a	1262 ± 262a
57142	988 ± 287a	1344 ± 283b
p-value	0,005**	0,02*
H×D p-value	0,18	0,11

*Significant at 5%; ** highly significant at 1%; *** very highly significant at 0.1%; numbers followed by the same letters in the same column are not statistically different at the 5% level; H×D: interaction between ridge height and planting density.

The increase in planting density within 15 cm high ridges results in yield increase of 113% and 118% for fruit with and without crowns, respectively. Similar observations were noted for ridges of 30 cm and 45 cm; as planting density increased to 57,142 plants.ha^-1^, the yield of crowned fruits rose by 149% and 129%, respectively (**Tables 5**, **6**). Although 45 cm ridges produce the highest yields, it is recommended to cultivate on 30 cm ridges, as the yield obtained from 30 cm ridges is not statistically different from that of 45 cm ridges, while offering higher economic profitability, with returns of 8.9 and 5.6 FCFA for densities of 27,777 and 57,142 plants.ha^1^, respectively (**Table 7**). The yield obtained under zero tillage in waterlogged soil conditions is 14,638 ± 2,140 kg.ha^1^, which is 1.8 times lower than that obtained on 15 cm ridges. Each 1 CFA invested in ridge formation generates profits of 5, 6, and 4 FCFA for ridges of 15, 30, and 45 cm at a density of 27,777 plants.ha^1^ (**Table 7**), and 11, 14, and 10 FCFA for the same respective ridge heights at a density of 57,142 plants.ha^1^ (**Table 8**). Investing in ridge formation enhances both technical and economic performance for producers operating in waterlogged or compacted soil conditions. However, planting density enhances economic performance twofold compared to ridge formation. The lowest profitability is associated with 45 cm ridges, which should only be recommended if the water level exceeds 15 cm above the soil surface.

**Table 5:** Effect of ridge height and planting density on yield of pineapples without crowns.

Ridge height	Plant density (plants.ha^-1^)	
	27,777	57,142
	Yield (kg.ha^-1^)	
15 cm	16315 ± 638aA	35652 ± 2975aB
30 cm	26316 ± 3550bA	65466 ± 1086bB
45 cm	29894 ± 3223bA	68310 ± 10122bB

The numbers followed by lowercase letters in the same column are not statistically different at the 5% level; the numbers followed by uppercase letters in the same row are not statistically different at the 5% level.

**Table 6:** Effect of ridge height and planting density on crowned pineapple yield.

Ridge height	Plant density (plants.ha^-1^)	
	27,777	57,142
	Yield (kg.ha^-1^)	
15 cm	26198 ± 912aA	55663 ± 3704cB
30 cm	37274 ± 3363bA	86951 ± 1115dB
45 cm	41706 ± 3549bA	87780 ± 6945dB

The numbers followed by lowercase letters in the same column are not statistically different at the 5% level; the numbers followed by uppercase letters in the same row are not statistically different at the 5% level.

**Table 7:** Economic profitability of the combination of ridge height and planting density based on the reference density.

RH (cm)	PD (plants.ha^-1^)	Ye (kg.ha^-1^)	AFW (g)	ASWC	APDC	AHC	TIC	AHR	IRC	BCR	PR
0	27777	14638	0,527	0	0	/	/	/	/	/	/
15	27777	26198,3	0,943	111488	0	0	111488	919269	116226	6	5
30	27777	37274,4	1,342	214400	0	0	214400	2108589	223512	7	6
45	27777	41706,6	1,501	335321	0	0	335321	2253493	349572	5	4
15	57142	55663,5	0,974	111488	587300	58730	757518	3158640	789713	3	2
30	57142	86951,1	1,522	214400	587300	58730	860430	5940285	896998	5	4
45	57142	87779,6	1,536	335321	587300	58730	981351	5951635	1023058	4	3

RH, ridge height; PD, planting density; Ye, yield; AFW, average fruit weight; ASWC, additional soil working cost; APDC, additional density cost; AHC, additional harvesting cost; TC, total investment cost; AHR, additional harvest revenue; IRC, investment return cost; BCR, benefit cost ratio; PR, profitability.

**Table 8:** Economic profitability of the combination of ridge height and planting density based on varying densities.

RH (cm)	PD (plants.ha^-1^)	Ye (kg.ha^-1^)	AFW (g)	ASWC	APDC	TIC	AHR	IRC	BCR	PR
0	27777	14638	527	0						
15	27777	26198	943	111488	43872	155360	919269	161963	6	5
30	27777	37274	1341	214400	85906	300306	2108589	313069	7	6
45	27777	41707	1501	335321	102727	438048	2253493	456665	5	4
0	57142	30113	527	0						
15	57142	55664	974	111488	52458	163946	1967198	170914	12	11
30	57142	86951	1521	214400	74705	289105	4669077	301392	15	14
45	57142	87780	1536	335321	75078	410399	4692424	427841	11	10

RH, ridge height; PD, planting density; Ye, yield; AFW, average fruit weight; ASWC, additional soil working cost; APDC, additional density cost; AHC, additional harvesting cost; TIC, total investment cost; AHR, additional harvest revenue; IRC, investment return cost; BCR, benefit cost ratio; PR, profitability.

## 4 Discussions

### 4.1 Impact of waterlogging on aboveground and belowground growth and development of pineapple

The high risk of waterlogging is primarily due to the accumulation of clay and silt in the deeper soil horizon (30–48 cm, ABtC horizon), which restricts water infiltration. This risk is influenced more by physical soil characteristics than by the annual amount of rainfall. Similar observations were reported by Djukem and Nkouathio (2023) in the Mbo Plain, located in the Littoral Region of Cameroon. The slow growth of pineapple observed on the 15 cm ridge height and in the surrounding zero-tillage plot indicates that pineapple has limited tolerance to waterlogged soil conditions. The resulting hypoxic conditions impair root functioning and reduce water movement into the plant, which disrupts stomatal regulation and limits CO_2_ uptake by the plant. These results are consistent with findings from previous studies of Cahyono et al. (2018), who observed that pineapple roots under temporary waterlogged conditions of more than 72 hours fail to develop absorbent root hairs, indicating that pineapples are more tolerant to drought than to hydromorphic conditions. Min and Bartholomew (2002) reported that waterlogging significantly reduces CO_2_ fixation and utilization in pineapple, leading to decreased photosynthetic capacity and, consequently, lower production and translocation of assimilates. Furthermore, the recovery of photosynthetic potential after prolonged exposure to waterlogged conditions is limited and often irreversible. In contrast, a ridge height of 30 and 45 cm promotes optimal CO_2_ fixation and nutrient uptake due to reduced hypoxic stress in the root zone promotes optimal growth and higher D-leaf surface area between 150- and 240-DAP. This outcome is consistent with studies by Lee et al. (2015); Padonou et al. (2019) and Tay (1974).The growth differential observed between plants on 30 cm and 45 cm ridges aligns well with findings by Li et al. (2022), who reported that a reduced contact area between soil-root complexes limits water and nutrient absorption, a phenomenon typically occurring at depths exceeding 40 cm. Additionally, Ogban and Babalola (2002) showed that 45 cm ridges exhibit higher bulk density and reduced aeration compared to lower soil layers, due to the greater compaction at the 45 cm ridges, a capillary bound often forms, altering water movement along the ridge profile. Ma et al. (2024) highlighted that capillary rise is limited to the top 25 cm, with lower hydraulic conductivity in the 25 to 42 cm range on ridges, suggesting a capillary bound that affects water movement within the soil. This barrier accelerates water saturation at the ridge top, thereby explaining the reduced growth of pineapples on 45 cm ridges compared to those on 30 cm ridges in rainy season. Observations of longer and wider fruits on 45 cm ridges also align with Tay (1974), who show that, the average weight of fruits on soils with a water table at the surface is 0.33 kg. This average fruit weight increases by 233.3%, 309.1%, and 318.2%, respectively, when the water table is at depths of 18, 33, and 48 cm. Incorporating soil moisture sensors, as noted in recent studies, could optimize water management and prevent water stress, contributing to more stable yield outcomes.

### 4.2 Ridge tillage management practice optimize yield of pineapple under waterlogged condition

The fruit yield of 86.9 tons.ha^-1^ observed on 30 cm ridges at a planting density of 57,142 plants per hectare, without fertilization, is 26% higher than the yield reported under optimal fertilization conditions in Benin at the same planting density (Djido et al., 2021). This yield represents 83% of the yield achieved in Peru under comparable planting densities (Neri et al., 2021). Water availability throughout the pineapple growth cycle is crucial for achieving medium-sized fruits (grades G to E according to Codex STAN 180-1993). Pineapple is highly sensitive to both water shortage and water excess, primarily due to its shallow root system (Cahyono et al., 2018; Sossa et al., 2020). Planting density does not affect fruit masse at all ridge height this result is aligning with the findings from Djido et al. (2021) and Neri et al. (2021). The number of active leaves, a physiological trait, is not directly influenced by agronomic practices but rather by local climatic conditions, supporting conclusions from Djido et al. (2021). The observed 6% (82 g) increase in crown fruit weight contrasts with previous reports, which indicate a decrease of 100 g per fruit with every 10,000 plants.ha^1^ increase in density (Valleser, 2018) or show no effect of density on fruit weight (Djido et al., 2021; Neri et al., 2021). However, under conditions of adequate water availability, higher densities enhance resource utilization and increase fruit weight. Studies by Tian et al. (2017) demonstrate that high-density planting improves resource use efficiency, reducing nitrogen requirements by 33% and increasing the number of tillers by 3% to 12%. Each kilogram of nitrogen can be replaced by increasing rice planting density by 1,000 plants per hectare. These findings prompt a critical evaluation of whether current soil fertility management practices in pineapple-based systems are sustainable for long-term use. Including precision fertilization techniques and denser plant configurations could further refine the sustainable yield benefits seen with current ridge tillage practices. Ridge formation in general improves aeration in waterlogged conditions and enhances water retention during dry periods through capillary uplift, with ridges of 30 cm showing major effects in this study. Additionally, the concentration of arable soil within the ridge optimizes rhizosphere nutrient availability to pineapple. Increased root–soil contact resulting from higher planting densities enhances resource uses efficiency without adversely affecting growth parameters. The close proximity of roots at high planting densities increases the overall suction force of plants in the soil through a form of synergy created by the interconnected root network. Similarly, the joint synthesis of root exudates raises their concentration and more strongly alters the chemical composition of the rhizosphere, thereby enhancing root water and nutrient uptake (Cooper et al., 2018; Landl et al., 2021). This explains why the interaction between ridge height and high planting density results in higher yields compared to lower planting densities.

## 5 Conclusion

The research findings affirm that both ridge height and planting density significantly influence the growth and yield of pineapple plants under waterlogged soil conditions. Specifically, ridge heights of 30 cm combined with higher planting 57,142 plants.ha^-1^ densities yielded optimal results, suggesting a synergistic effect that enhances fruit quality and economic profitability. The observed increase in fruit weight and yield underscores the necessity for targeted agronomic practices to optimize resource utilization, thereby contributing to sustainable agricultural systems. Given the adverse effects of waterlogging on pineapple performance, the adoption of ridge cultivation and appropriate planting density can serve as effective strategies to mitigate these challenges and enhance the viability of pineapple production. Future research should focus on long-term sustainability and the economic implications of these practices in diverse environmental conditions.

## Data Availability

The raw data supporting the conclusions of this article will be made available by the authors, without undue reservation.

## References

[B1] Barker D. L. Arantes S. D. Schmildt E. R. Arantes L. de O. Fontes P. S. F.. (2018). Post-harvest quality of ‘Vitória’ pineapple as a function of the types of shoots and age of the plant for floral induction. Rev. Bras. Frutic. 40, 1–13. doi: 10.1590/0100-29452018297

[B2] Beegum S. Truong V. Bheemanahalli R. Brand D. Reddy V. Reddy K. R. (2023). Developing functional relationships between waterlogging and cotton growth and physiology-towards waterlogging modeling. Front. Plant Sci. 14, 1174682. doi: 10.3389/fpls.2023.1174682 37583596 PMC10425224

[B3] Cahyono P. Purwito Afandi (2018). Effects of waterlogging on pineapple growth and soil properties on red acid soils of lampung, Indonesia. Available online at: http://repository.lppm.unila.ac.id/id/eprint/11512 (Accessed December 25, 2023).

[B4] Cooper L. J. Daly K. R. Hallett P. D. Koebernick N. George T. S. Roose T. (2018). The effect of root exudates on rhizosphere water dynamics. Proc. R. Soc A. 474, 20180149. doi: 10.1098/rspa.2018.0149 30333700 PMC6189581

[B5] Djido U. Fassinou Hotegni N. V. Lommen W. J. M. Hounhouigan J. D. Achigan-Dako E. G. Struik P. C. (2021). Effect of planting density and K2O:N ratio on the yield, external quality, and traders’ Perceived shelf life of pineapple fruits in Benin. Front. Plant Sci. 12. doi: 10.3389/fpls.2021.627808 PMC8244590 34220877

[B6] Djukem F. S. N. Nkouathio D. G. (2023). Contribution of soil physical properties in the assessment of flood risks in tropical areas: case of the Mbo plain (Cameroon). Nat. Hazards 116, 3447–3463. doi: 10.1007/s11069-023-05818-0

[B7] Dos Santos M. P. Maia V. M. Oliveira F. S. Pegoraro R. F. Dos Santos S. R. Aspiazú I. (2018). Estimation of total leaf area and d leaf area of pineapple from biometric characteristics. Rev. Bras. Fruticultura 40, 1–4. doi: 10.1590/0100-29452018556

[B8] Etame Kossi G. M. Beyegue D. H. Boukong A. Silatsa Tedou F. B. (2023a). Extensive pineapple production constraints and land suitability in the centre region of Cameroon. Agric. Sci. 14, 240–255. doi: 10.4236/as.2023.142016

[B9] Etame Kossi M. G. Djonko H. B. Chotangui A. H. Boukong A. Awono J.-P. M. (2023b). Typologies of pineapple-based farming systems in Centre-Cameroon. AJAR 19, 247–259. doi: 10.5897/AJAR2022.16083

[B10] Ferreira E. B. Cavalcanti P. P. Nogueira D. A. (2014). ExpDes: an R package for ANOVA and experimental designs. Appl. Mathematics 5, 2952. doi: 10.4236/am.2014.519280

[B11] Firatoiu A.-R. Chiurciu I.-A. Marcuta L. Chereji A.-I. Soare E. Voicu V.. (2021). “Study on the production and marketing of pineapples worldwide,” in Proceedings of the 37th international business information management association (IBIMA). (Cordoba, Spain: IBIMA), 1–2.

[B12] Fox J. Muenchen R. Putler D. Fox M. J. (2023). Package ‘RcmdrMisc.’. Available online at: http://mirrors.nic.cz/R/web/packages/RcmdrMisc/RcmdrMisc.pdf (Accessed October 30, 2024).

[B13] Jagoret P. Michel-Dounias I. Snoeck D. Ngnogué H. T. Malézieux E. (2012). Afforestation of savannah with cocoa agroforestry systems: A small-farmer innovation in central Cameroon. Agroforestry Syst. 86, 493–504. doi: 10.1007/s10457-012-9513-9

[B14] Jones A. Breuning-Madsen H. Brossard M. Chapelle J. Dampha A. Deckers J.. (2015). Atlas des sols d’Afrique. (Luxembourg: Publié par le Bureau des publications de l’Union européenne, L-2985 Luxembourg) 1–176.

[B15] Landl M. Phalempin M. Schlüter S. Vetterlein D. Vanderborght J. Kroener E.. (2021). Modeling the impact of rhizosphere bulk density and mucilage gradients on root water uptake. Front. Agron. 3, 622367. doi: 10.3389/fagro.2021.622367

[B16] Lee S. W. Lee S. H. Jang I. B. Lan J. M. Park K. H. Kim K. H. (2015). Effect of ridge height on growth characteristics and yield of 6 year old Panax ginseng in cultivation of paddy soil. Korean J. Medicinal Crop Sci. 23, 351–356. doi: 10.7783/KJMCS.2015.23.5.351

[B17] Li H. Li L. Liu N. Chen S. Shao L. Sekiya N.. (2022). Root efficiency and water use regulation relating to rooting depth of winter wheat. Agric. Water Manage. 269, 107710. doi: 10.1016/j.agwat.2022.107710

[B18] Ma L. Wang S. Ni C. Wang W. Kang S. Li Z.. (2024). Measurement and modelling of soil water dynamics under ridge tillage in paddy field. Soil Tillage Res. 244, 106172. doi: 10.1016/j.still.2024.106172

[B19] Manik S. M. N. Pengilley G. Dean G. Field B. Shabala S. Zhou M. (2019). Soil and crop management practices to minimize the impact of waterlogging on crop productivity. Front. Plant Sci. 10. doi: 10.3389/fpls.2019.00140 PMC6379354 30809241

[B20] Min X.-J. Bartholomew D. P. (2002). Effects of flooding and drought on ethylene metabolism, titratable acidity and fruiting of pineapple. IV Int. Pineapple Symposium 666, 135–148. doi: 10.17660/ActaHortic.2005.666.13

[B21] Neri J. C. Meléndez Mori J. B. Vilca Valqui N. C. Huaman E. Collazos Silva R. Oliva M. (2021). Effect of planting density on the agronomic performance and fruit quality of three pineapple cultivars (Ananas comosus L. Merr.). Int. J. Agron. 2021, 1–9. doi: 10.1155/2021/5559564

[B22] Ogban P. I. Babalola O. (2002). Evaluation of drainage and tillage effect on watertable depth and maize yield in wet inland valleys in southwestern Nigeria. Agric. Water Manage. 52, 215–231. doi: 10.1016/S0378-3774(01)00135-4

[B23] Padonou G. E. Aholoukpe H. N. S. Sossa E. L. Saidou A. Amadji G. L. (2019). Réponse de l’ananas (Ananas comosus L. Merrill) à la fertilisation minérale élémentaire sur sol ferrallitique au Sud du Bénin. Int. J. Biol. Chem. Sci. 12, 2653. doi: 10.4314/ijbcs.v12i6.15

[B24] Sossa E. L. Hounsou B. M. Codjo E. (2020). Influence of tillage and mulching on soil water balance under pineapple crop (Ananas comosus (l) Merr). Int. J. Of Eng. Res. And Appl. 10, 01–09. doi: 10.9790/9622-1010010109

[B25] Tay T. H. (1974). Effect of water on growth and nutrient uptake of pineapple. Bull 2, 39–41.

[B26] Tian G. Gao L. Kong Y. Hu X. Xie K. Zhang R.. (2017). Improving rice population productivity by reducing nitrogen rate and increasing plant density. PloS One 12, e0182310. doi: 10.1371/journal.pone.0182310 28767723 PMC5540556

[B27] Tian L. Zhang Y. Chen P. Zhang F. Li J. Yan F.. (2021). How does the waterlogging regime affect crop yield? A global meta-analysis. Front. Plant Sci. 12, 634898. doi: 10.3389/fpls.2021.634898 33679848 PMC7933672

[B28] Tyagi A. Ali S. Mir R. A. Sharma S. Arpita K. Almalki M. A.. (2024). Uncovering the effect of waterlogging stress on plant microbiome and disease development: current knowledge and future perspectives. Front. Plant Sci. 15, 1407789. doi: 10.3389/fpls.2024.1407789 38903424 PMC11187287

[B29] Vallerie M. (1973). Contribution a l’etude des sols du centre sud cameroun types de differenciation morphologique et pedogenetique sous climat subequatorial. O.R.S.T.O.M. (ORSTOM Paris).

[B30] Valleser V. C. (2018). Planting density influenced the fruit mass and yield of ‘Sensuous pineapple. Int. J. Sci. Res. Publications (IJSRP) 8, 113–9. doi: 10.29322/ijsrp.8.7.2018.p7919

